# A Simple Approach for Molecular Controlled Release based on Atomic Layer Deposition Hybridized Organic-Inorganic Layers

**DOI:** 10.1038/srep19574

**Published:** 2016-01-21

**Authors:** Christian Boehler, Firat Güder, Umut M. Kücükbayrak, Margit Zacharias, Maria Asplund

**Affiliations:** 1 Freiburg Institute for Advanced Studies (FRIAS), Albert-Ludwigs-Universität Freiburg, 79104 Freiburg, Germany; 2 Laboratory for Biomedical Microtechnology, Department of Microsystems Engineering (IMTEK), University of Freiburg, 79110 Freiburg, Germany; 3 Laboratory for Nanotechnology, Department of Microsystems Engineering (IMTEK), University of Freiburg, 79110 Freiburg, Germany

## Abstract

On-demand release of bioactive substances with high spatial and temporal control offers ground-breaking possibilities in the field of life sciences. However, available strategies for developing such release systems lack the possibility of combining efficient control over release with adequate storage capability in a reasonably compact system. In this study we present a new approach to target this deficiency by the introduction of a hybrid material. This organic-inorganic material was fabricated by atomic layer deposition of ZnO into thin films of polyethylene glycol, forming the carrier matrix for the substance to be released. Sub-surface growth mechanisms during this process converted the liquid polymer into a solid, yet water-soluble, phase. This layer permits extended storage for various substances within a single film of only a few micrometers in thickness, and hence demands minimal space and complexity. Improved control over release of the model substance Fluorescein was achieved by coating the hybrid material with a conducting polymer film. Single dosage and repetitive dispensing from this system was demonstrated. Release was controlled by applying a bias potential of ±0.5 V to the polymer film enabling or respectively suppressing the expulsion of the model drug. *In vitro* tests showed excellent biocompatibility of the presented system.

Controlled delivery of bioactive substances with local restriction is of interest in medicine and life sciences, covering a large field from basic research to practical applications including lab-on-a-chip systems^1^,^2^,^3^,^4^. For this purpose, spatial and temporal control of release is as important as the possibility to precisely adjust the amount of released substances which can be anything from small-sized ions to large molecules or proteins. Different applications demand different properties of the release systems which can generally be categorized according to their functionality into passively or actively eluting systems. Active systems can furthermore be separated into direct systems with storage and release functionality combined in one single material, hence being small and compact, or indirect systems which only provide the control and rely on an external drug supply i.e. valve-like structures in microfluidic devices^5^.

A promising approach to cover relevant requirements for an active implantable release system employing conducting polymers has recently been proposed^1^,^6^,^7^. This class of materials features the possibility to incorporate and release ionic substances on demand, and accordingly acts as a direct release system with a compact architecture. Various studies report successful release of molecules such as Dex, NGF, neurotransmitters or BDNF from polymers like poly(3,4-ethylenedioxythiophene) (PEDOT) or polypyrrole (PPy)^8^,^9^,^10^,^11^,^12^. However, the amount of substance that can be entrapped, and hence released, is restricted to small molecules and the limited storage capability of the thin polymer film^13^. In addition, the polymer film is vulnerable to delamination and cohesive failure when the film thickness exceeds several micrometers^14^,^15^. Studies employing co-deposition processes for realization of nano-pores or layered structures to increase the drug load demonstrated enhanced release properties compared to simple polymer systems^16^,^17^,^18^,^19^,^20^. Nevertheless, these approaches are still restricted by the overall properties of the conducting polymer, limiting the possibility to realize thick and thus highly loaded films.

In this study we present a new approach for the fabrication of a general release system, based on the separation of storage and release functionality for controlled delivery, yet retaining simplicity. The design benefits from the exceptional properties of a conducting polymer in actively controlling the transmission of molecules through the polymer matrix upon redox activation. In other words, the porosity of the polymer film can be actively modulated to allow release of the underlying molecules. Insufficient storage properties of the polymer are targeted by the design of a new hybrid material based on polyethylene glycol (PEG) and ZnO. Both materials are well characterized and known to be biocompatible. While the PEG serves as dispensing medium for potentially any kind of biologically relevant molecules, the subsequent atomic layer deposition (ALD) of ZnO converts the liquid drug-solution into a solid hybrid layer, forming the storage phase. This solid film can be coated with a thin conducting polymer film serving as gate-keeper, enabling an active release system for a broad variety of substances. We thereby demonstrate the feasibility of converting a drug-containing liquid polymer into a solid hybrid material with ALD. This is to the best of our knowledge the first time ALD has been used to induce a phase transformation in a liquid substrate. Furthermore we demonstrate the successful storage and release functionality using fluorescein as a model substance.

## Results

### Hybridization of liquid polymers by ALD

Atomic layer deposition (ALD) is an emerging thin film deposition technique which allows growth of highly conformal thin films on a variety of substrates with sub-nm thickness control. Unlike conventional chemical vapor deposition (CVD), layer growth is achieved by releasing the gas phase precursors into the reaction chamber in a sequential manner which permits deposition of a large range of materials such as oxides, nitrides, metals etc. in a wide temperature window including room temperature^21^,^22^,^23^,^24^. These features of ALD make it an attractive option for conformal thin film growth on temperature sensitive substrates.

**Figure 1:**
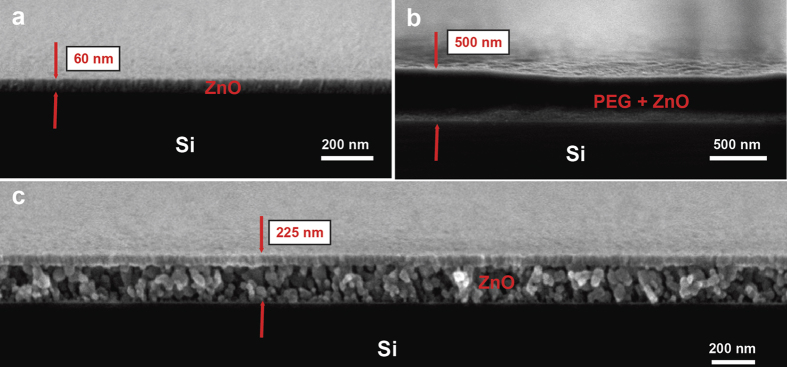
(**a**) Cross-sectional electron micrograph of 300 cycle ALD ZnO thin film deposited on a bare Si substrate at 150 °C; (**b**) ALD hybridized PEG-ZnO thin film. The process conditions were the same as (**a**); (**c**) The same film as shown in b after annealing at 500 °C in air.

Figure 1a shows a scanning electron microscopy (SEM) image of a ZnO ALD layer grown on a bare Si substrate. The ALD process consisted of 300 deposition cycles at 150 °C with diethylzinc (DEZ) and deionized water as respective reactants and yielded a thickness of 60 nm.

From the figure, it can be seen that the resulting film is uniform and has a polycrystalline microstructure. When the same process was repeated to deposit a ZnO layer on a PEG 400 coated Si wafer, the resulting structure was significantly different as can be seen in Fig. 1b. The ZnO layer was once again present on top of the spin coated polymer, however, this time slightly thinner (~50 nm). The PEG 400 film, which is a liquid polymer at room temperature, was converted into a solid film. In order to study this phenomenon, the polymer was burnt away at 500 °C for one hour in air. Figure 1c shows an electron micrograph of the sample after the annealing process. The remaining layer was a 225 nm thick low density granular thin film, as opposed to the original film thickness of 500 nm, and was comprised of ZnO particles. The top 50 nm displayed a higher density of the ZnO which appeared similar to the original ZnO ALD film as shown in Fig. 1a. Interestingly, the ALD precursors were able to penetrate deep into the PEG layer and reacted to form ZnO within the bulk of this gel like film instead of forming a 60 nm ZnO film on the surface of the polymer. As these large particles were not visible in Fig. 1b, they must have coalesced into their current size from much smaller nanoscale particles during the thermal decomposition of the PEG matrix. Basically, by coating a liquid polymer such as PEG 400 with the ALD process, we were able to create a hybridized organic-inorganic solid thin film. In addition, this technique represents a new strategy to form various porous thin films such as the one shown in Fig. 1c.

**Figure 2:**
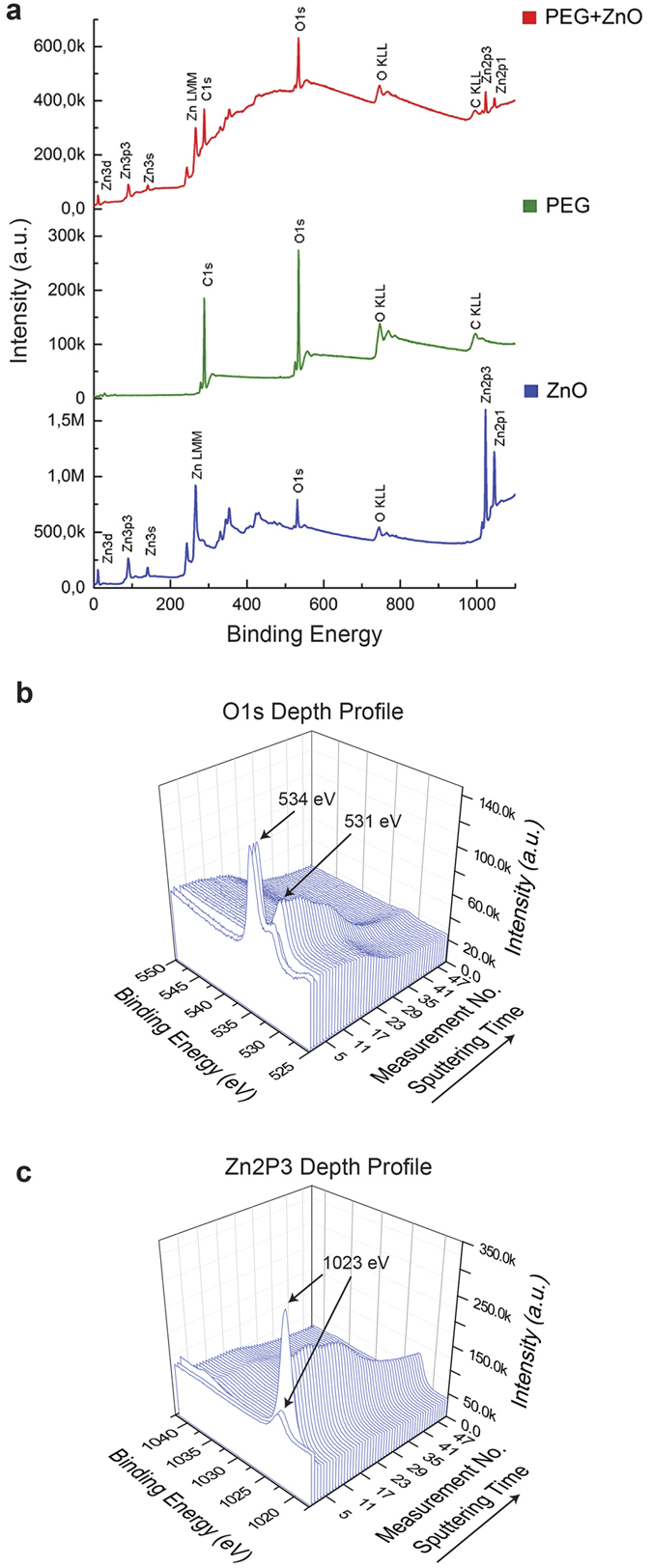
(**a**) X-ray photoelectron spectroscopy survey of all three prepared samples namely, bare ZnO, bare PEG and the hybridized thin film; (**b**) Detailed spectra of the O1s and; (**c**) Zn2p3 peaks for hybridized PEG-ZnO thin films with respect to sputtering time (i.e. depth).

X-ray photoelectron spectroscopy (XPS) analysis was performed to further investigate this layer. The XPS spectral surveys of the hybridized PEG-ZnO, ALD ZnO, and a spin coated PEG layer are shown in Fig. 2a. Studying the Zn2p3 peaks, especially comparing the ZnO layer grown on Si and the hybridized layer, we could not identify any peak shifts between these two samples. Hence, Zn existed in the same chemical state in both samples. However, the intensity of the signal was several times smaller for the hybridized film.

Furthermore, comparing the O1s signal for the different samples, it could be confirmed that the position of the O1s peak in the hybridized film was the same as for the pure PEG sample. These two clues indicate that an ALD film growth took place not only on the surface but also in the sub-surface region of the polymer film. In order to confirm this finding, we performed XPS depth profiling by sputtering the sample surface with Ar atoms; the results are shown in Fig. 2b,c. It can clearly be seen that, as the sputtering time increases, the O1s peak shifts towards the position recorded from the ALD ZnO sample (Fig. 2b). The peak at 534 eV results from moisture (H_2_O) adsorbed on the sample surface and thus is only detected initially in the depth profile. As for the Zn2p3 peak, the signal intensity initially increases with progressing sputtering time and then gradually decreases, showing that the ZnO distribution is not uniform throughout the layer.

### Release from hybridized ZnO-PEG films

We synthesized drug loaded hybrid storage films containing fluorescein as a model substance by blending the molecule into the PEG and subsequently converting the polymer into a hybrid solid material using an ALD process. In order to increase the film adhesion we used porous Si as the substrate. The porous Si was fabricated by the well-established metal assisted chemical etching strategy using Ag as the catalyst^25^,^26^. This simple technique improved substrate adhesion of the hybridized layer by enabling mechanical interlocking at the interface.

**Figure 3:**
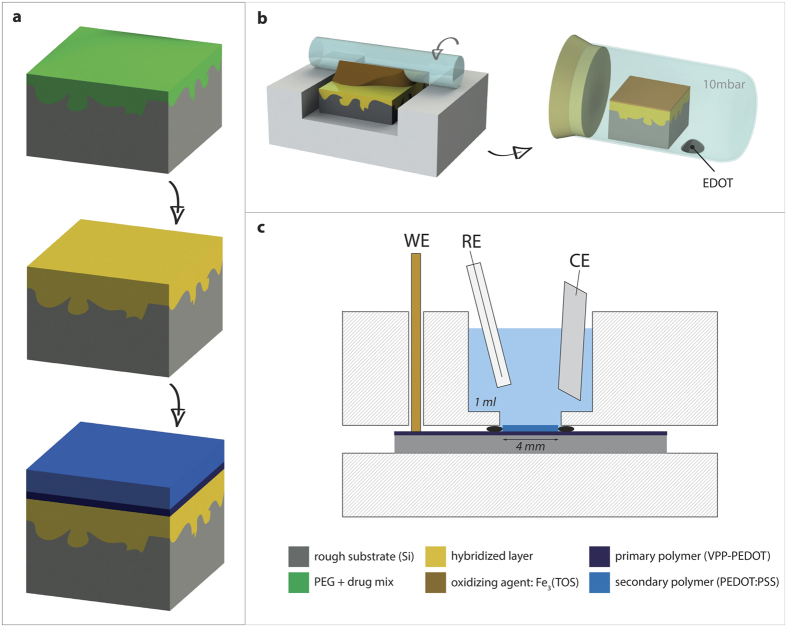
Fabrication of hybridized release samples. (**a**) Spin-coating of a drug-containing PEG film (green) onto the substrate, followed by the hybridization (ZnO-ALD for defining the storage part shown in “yellow”). Subsequent coating with conducting polymer layers (blue) builds the complete release system. (**b**) Process scheme for fabrication of the primary polymer layer using the vapor phase polymerization. First the oxidizer is applied in a roll-on technique, followed by the polymer formation in a vacuum-chamber. (**c**) Illustration of the three electrode setup used for the electrochemical deposition of the secondary polymer or for application of the bias potential during the release studies.

The hybridization of the drug loaded film during ALD did not show any difference to the pure PEG/ZnO samples, confirming independence of the solidification process from the loaded substance. It should be noted that if the loaded substance is highly reactive with the ALD precursor, secondary reactions might alter the chemical properties of the loaded molecules. This needs to be addressed individually for the target molecule of interest. Nevertheless, the drug will be mostly protected by the liquid. After completing the formation of the storage film (Fig. 3a), a conducting polymer layer (primary polymer) was deposited using the vapor-phase deposition procedure shown in Fig. 3b. The growth of this layer from the gas phase enabled capping of the storage film without compromising the integrity of the hybrid material. A subsequent color change from orange (oxidizer) to black (polymer) could be observed in the vacuum chamber during the chemical process, indicating the formation of the conducting polymer.

**Figure 4:**
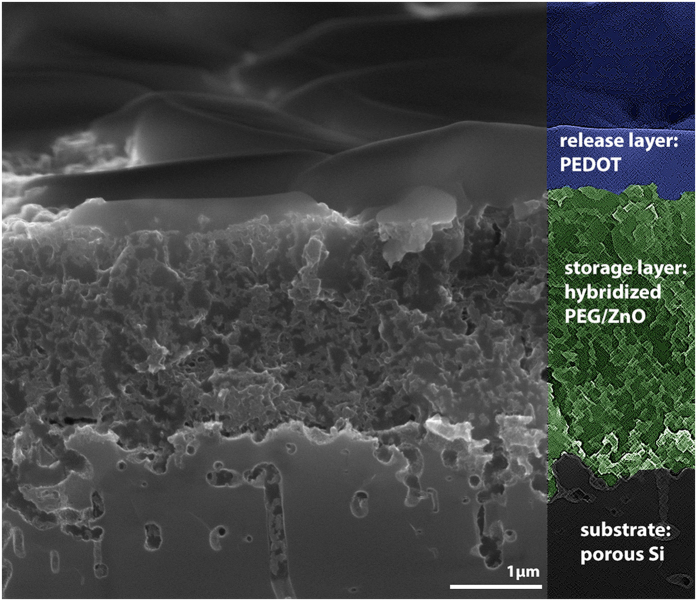
Cross-sectional Scanning Electron Microscopy (SEM) image of the release system. The hybridized storage layer is marked in green (middle) with the primary conducting polymer layer on top, represented in blue.

This layer, having a thickness of roughly 1 μm, did not only serve as a capping layer for the protection of the water-sensitive PEG/ZnO film but also added the possibility to electrically contact the sample. A resistivity value of less than 10 kΩ (compared to > 10 MΩ for the hybrid material) could be accomplished across a sample of 1 cm^2^ which proved sufficient for subsequent processing. Accordingly a secondary polymer layer could be deposited using an electro-polymerization process in a conventional three electrode setup (Fig. 3c) in order to grow a dense and well characterized polymer layer for precise control of the release functionality. An SEM image showing the complete stack of layers after deposition of the VPP polymer is provided in Fig. 4. All three layers representing the porous substrate (grey, bottom), the storage layer (green, middle) and the primary polymer (blue, top) are clearly visible. A dense integration of the separate films at the corresponding interfaces is apparent. Moreover, it can be seen that the vapor phase deposition procedure enabled the formation of the conducting polymer not only on top of the hybrid film but also within the pores of the top layer.

**Figure 5:**
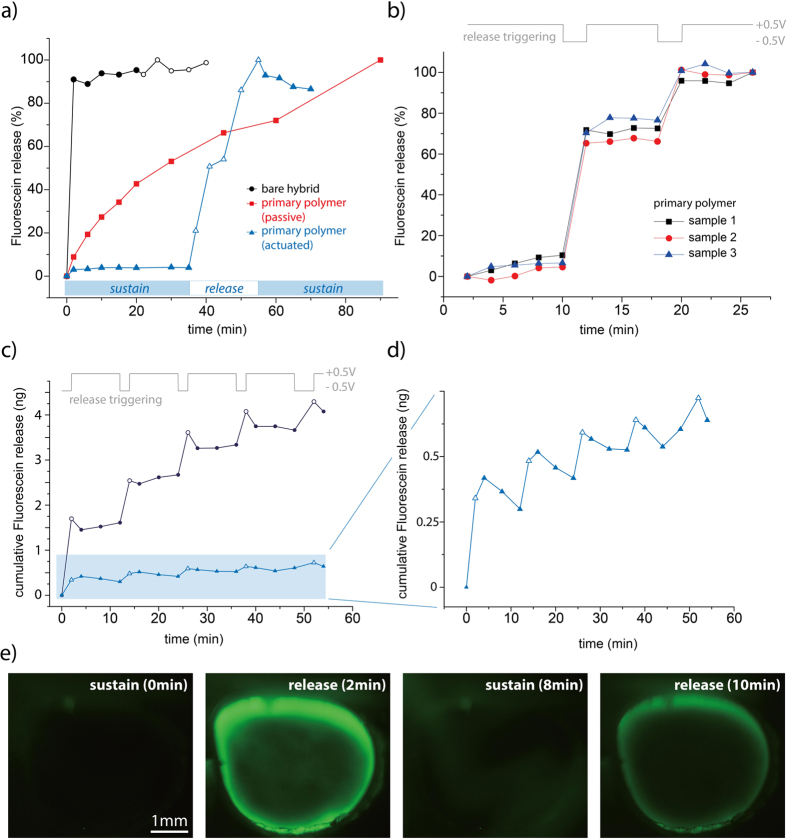
Release results using fluorescein as model substance. (**a**) Comparison of a bare hybrid sample (black circles) with a polymer-coated sample that is not electrically contacted (red squares) and a corresponding sample having a bias potential (blue triangles). (**b**) Multiple dosage behavior of three individual samples with the primary polymer. (**c**) Comparison of a multiple release system with the primary polymer (black circles) and the secondary polymer (blue triangles) (**d**) Magnification of the secondary polymer sample shown in (**c**). (**e**) Fluorescence imaging during subsequent release triggering.

Release functionality of the films was assessed by immersing the samples in saline solution and quantifying release by fluorometry. A bias potential of either −0.5 V or +0.5 V was applied to the release layer for triggering the fluorescein expulsion or suppressing the release respectively. Figure 5a shows a comparison of the main sample configurations during fabrication of the release system. The blue triangles represent a hybrid film, coated with the primary release layer and immersed under biased conditions, confirming the functionality of the system in single dispensing mode. As long as the positive bias potential was maintained, only an insignificant fluorescein release could be detected. By switching the potential to −0.5 V, however, substantial release of the model drug could be triggered. After 10 min at negative bias, the potential was reversed, resulting in stabilization of the fluorescent signal towards a constant value. Immediately, at the reversal of the potential, there was an abrupt drop in the signal, indicating that some of the fluorescein that was previously released was lost from solution upon switching polarity, probably due to re-uptake into the film.

The red squares in Fig. 5a represent a storage film having only the primary polymer coating (VPP-PEDOT) which was not electrically connected. One can identify a continuous leakage of the fluorescein, followed by a saturation behavior. This data demonstrated that the control functionality of the release samples does not follow a normally-closed behavior but requires a steady potential to completely sustain the fluorescein release. The black circles in Fig. 5a represent the bare hybrid PEG/ZnO film, showing an immediate disintegration of the hybrid storage layer in the presence of water. The use of a bias potential (either −0.5 V or +0.5 V) did not show any influence on this release behavior which is also the expected result considering the film itself is not expected to be electroactive. The sample, however, demonstrates the possibility to successfully store and release fluorescein from the hybrid material regardless of the additional PEDOT layer.

Figure 5b shows a repetitive release measurement for three individual samples with the primary polymer as gating layer. One can clearly see a correlation between the released mass and the trigger signal for the three samples. A standard deviation of less than 6% confirms that the technique is reproducible.

Figure 5c,d show a hybrid sample with primary and respectively secondary polymer under consecutive actuation, forming a multiple-shot system in contrast to the single-dosage behavior seen in Fig. 5a. The amount of released fluorescein correlates well with the release and sustain conditions given by the alternating potential. Furthermore, it is apparent that the secondary polymer layer provides a different release behavior, which enables smaller release steps, and provides higher controllability over the total amount of fluorescein in the storage layer. The secondary polymer displays an increased re-uptake of fluorescein after switching the potential in comparison to the primary polymer layer.

In addition to the analytical release measurements, fluorescence imaging was used for optical investigation of the release system. The results for two consecutive trigger events are presented in Fig. 5e, showing an intensive fluorescence signal for the active release compared to the sustain condition. The higher current density along the edge of the sample leads to the glowing ring effect which overexposes the image so that the release from the center of the 4 mm diameter sample cannot be visualized simultaneously. It should be mentioned that no crack formation or similar defects could be observed which confirms that release occurred through the intact polymer membrane and is not related to disintegration of the polymer layer. The rate of drug expulsion after reversing the potential from sustain to release condition was moderate, requiring 10–15 s before release could be visually confirmed (intensity continuously increased as long as the potential was maintained). It can be assumed that a substantial amount of fluorescein is needed to appear as a clearly visible green signal on the image and that release of low doses in the direct vicinity of the film is much faster than the visual confirmation.

**Figure 6:**
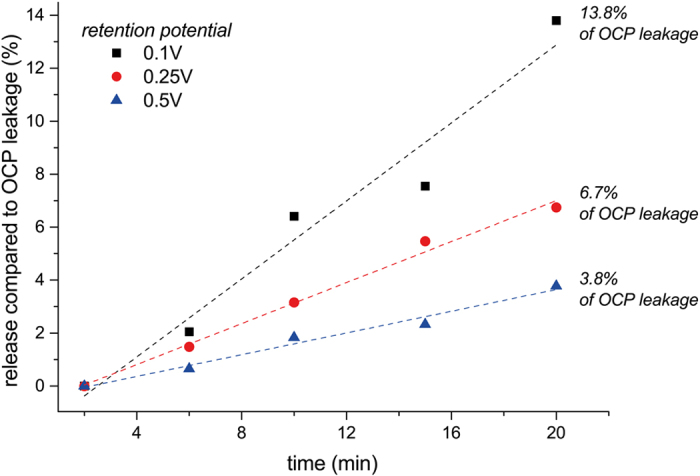
Influence of the retention potential on the leakage of the model drug with respect to the leakage of equivalent samples at open cell potential (OCP) during the same time-frame.

While the release potential was fixed at −0.5 V with the intention to allow maximal release efficiency and simultaneously avoid electrolysis of water, different retention potentials were tested with respect to their efficiency in suppressing the release from a thin VPP-polymer coated sample (Fig. 6). A linear correlation between the measured fluorescence intensity and the applied potential could be identified whereas the highest potential (+0.5V) showed the lowest leakage rate and thus the highest efficiency in suppressing the undesired release of the model drug.

Release efficiency of the coating was evaluated by applying a potential of −0.5 V for 30 min to the samples and measuring the total released mass of Fluorescein until the fluorescence intensity reached a saturation value as a consequence of the loading depletion. These values were subsequently compared to the theoretical loading of the film, which is calculated from the concentration of the model drug in the PEG (1 mg/ml) and the volume of the hybridized film (2.5 × 10^−5^ cm^3^), resulting in a loaded mass of 25 ng. The experimentally determined drug loading for the primary polymer sample was found to be 22 ng in average (n = 3) which corresponds to 88% of the calculated loading capacity. For the secondary polymer samples, the remaining drug loading, after all process steps, was found to be 20% of the theoretically loaded mass.

**Figure 7:**
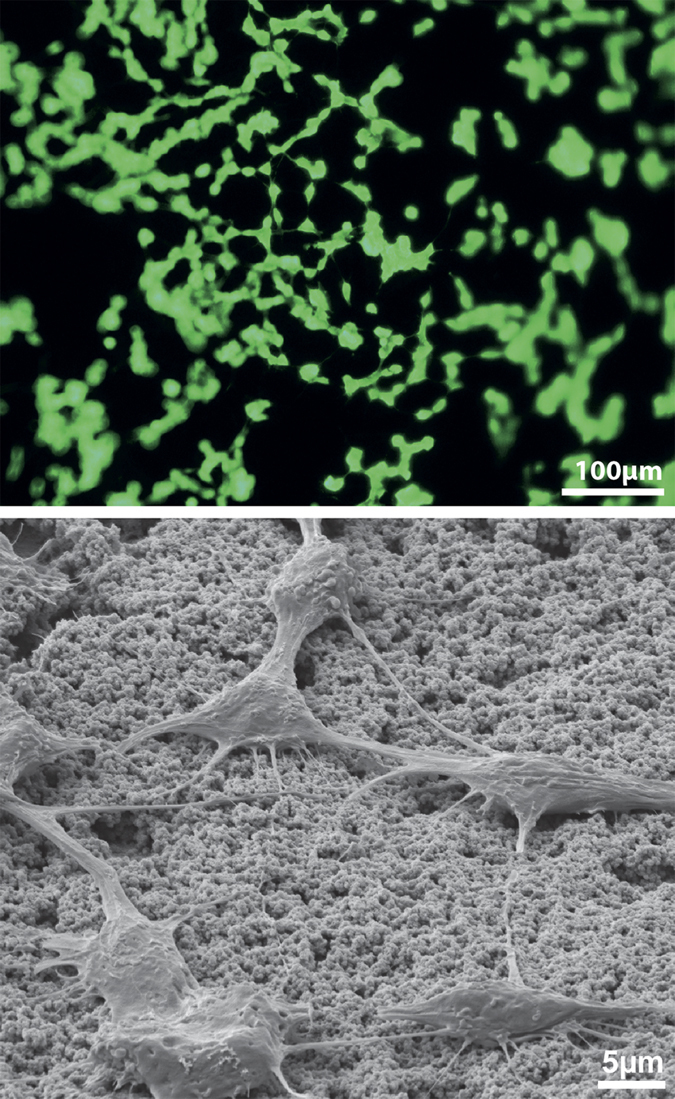
Biocompatibility testing of the hybrid material. (**a**) Densely spread SH-SY5Y cells sitting on the primary polymer of a hybrid release sample after 24 h exposure. Cells were able to incorporate passively released Fluorescein from the sample and are thus brightly fluorescent. (**b**) SEM imaging of the cells shown in (**a**) at 3000× magnification showing network formation and good integration with the polymer surface.

For biocompatibility evaluation of the system, *in vitro* tests were performed by culturing the neuron like cell line SH-SY5Y on top of a sample consisting of the hybridized PEG (including Fluorescein) and the primary polymer layer. After incubation for 24h, cells were fixed and imaged by fluorescence microscopy (Fig. 7a) as well as SEM (Fig. 7b). The pictures show homogenously spread cells growing adherently to the surface of the material. It was found that cells formed a network clearly demonstrating that the surface was suitable as a culture substrate. Besides these general biocompatibility aspects, we could also identify that the cells were able to incorporate the Fluorescein that was passively released from the hybrid storage film as seen in Fig. 7a.

## Discussion

In this paper, we successfully demonstrate the fabrication and functionality of a compact general release system by introducing a hybrid material based on PEG/ZnO. The storage and release functionality of the described system are separated into two different units enabling optimized design for both functions independently. The release functionality is formed by a conducting polymer (PEDOT), which has been shown to permit a change of the membrane permeability upon application of an electrical signal^27^,^28^. The proposed mechanism is that conformational changes of the polymer during redox, together with electrostatically driven ionic exchange, modulate the diffusion properties of the film. Water that penetrates the polymer film and elutes molecules from the storage layer accordingly provides free substances eligible for release under the polymer film. During subsequent activation of the conducting polymer, these free molecules are transported through the polymer matrix as a result of the aforementioned redox-properties of the polymer. This process is further assumed to be supported by a concentration gradient between the storage and the release side.

Fluorescein served as a model substance to characterize the release functionality of the formed system and it was shown that, by operating the system at negative or positive bias, the fluorescent substance could be efficiently contained or released on demand. Release was found to be distributed in proportion to the field distribution over the surface, with higher release at the edges. Release was not attributed to cracks or other defects of the film but appeared to be largely homogenous. Repetitive release/retain steps were employed to dispense multiple doses of fluorescein. The system response to such pulsed release was a reproducible step function which further confirms that the membrane remains intact and that the modulation of the permeability is reversible.

The membrane would per default be in the permeable state at open circuit. Thus, a continuous bias potential is required for maintaining the drug load and higher potential results in an improved retention efficiency. This is expected as a consequence of the higher oxidation state of the polymer. Maintaining a bias potential of 0.5 V requires a power consumption of 160 mW cm^−2^ for the sustain condition in comparison to 320 mW cm^−2^ (abs) for releasing the load. Assuming a battery capacity of 2 Ah, as provided by state of the art cardiac pacemakers, and adopting a suitable release area of 100 × 100 μm^2^, this would enable an operation time of >5 years on a 50% duty cycle. The permeability of the system could be influenced by forming a thicker gating layer by adding electropolymerized PEDOT on top of the VPP layer. Thus, the system response could easily be tuned to provide a larger or smaller quantity in response to the activation signal. A thicker gating layer is further expected to provide better retention efficiency under positive bias conditions compared to the thin gating layer shown in Fig. 6.

The slight signal drop immediately after reversing the potential presumably results from the re-uptake of fluorescein in the vicinity of the sample into the conducting polymer film. The ionic substance is thus electrostatically bound within the polymer film although not likely transported back to the storage layer. The amount of re-uptake is therefore limited by the thickness of the polymer film and the samples that are provided with an additional PEDOT layer (secondary layer) consequently display a stronger re-uptake upon reversal. Although not shown here, we expect that re-uptake could be suppressed either by facilitating diffusion away from the surface or by introducing an intermediate potential step, allowing more time for the substance to diffuse away from the surface, before a complete reversal of the bias is applied.

Conducting polymer based delivery films have frequently been reported by others^1^,^6^,^13^. These systems, however, lack a flexible storage possibility to provide the substance for release, relying only on what can be fitted in the conducting polymer film itself. Furthermore, the stability of the conducting polymer layer is negatively influenced by the loaded drug. A few studies describe release through conducting polymer based membranes^27^,^28^. Nevertheless, for drug supply, these systems require microfluidic devices or pumps to be connected to the release membrane, inevitably leading to elevated cost, complexity and size of any device.

In contrast to these approaches, the system presented here is simple, scalable and comparably cheap. We target the listed weaknesses by the introduction of an organic/inorganic hybrid layer that permits storage of different molecules (simultaneously) in large quantities directly at the release membrane. The storage layer thereby demands only a minimal amount of space and can conveniently be realized in no more than two process steps (spin-coating & ALD). The research groups of G. Parsons and S. M. George have extensively studied the dynamics of ALD coatings on polymer substrates^29^,^30^,^31^. They have concluded that polymers with non-reactive polymer backbones promote sub-surface ALD film growth. Although their results concern solid polymers, the same effect appears to apply to polymers that are in a liquid/gel-like state. In our case, the low molecular weight PEG with a non-reactive backbone, allows penetration of DEZ molecules deep into the layer and react with the OH groups attached to ends of the polymer chains. The subsequent water pulse reacts with the surface bound precursor and completes the ALD cycle. As sub-surface ALD growth progresses, an inorganic matrix is formed around the organic polymer enclosing it like a cage. We propose this is the reason why the liquid layer is converted to solid as a result of the here used ALD process.

Albeit the hybrid material was provided as flat layer on a silicon substrate in this study, it is not necessarily restricted to this shape or type of carrier. Both processes (ALD and vapor phase polymerization) are not limited to the wafer level and with a modified process it would be possible to realize layers on three dimensional structures like i.e. spheres or fibers. The simple fabrication, combined with the compact design and precise control over release, define the key features of the herein described release system.

We demonstrate functionality of the proposed system by characterizing the release of Fluorescein. Fluorescein was chosen as a model substance since it has a relevant size and charge in comparison to many drugs and because a strongly fluorescent molecule facilitates precise and fast detection. We would like to emphasize that fluorescein was only chosen as a model substance and the aim is delivery of bioactive substances. In principle, any molecule that is not negatively influenced by the ALD precursor and that remains thermally stable under the given processing conditions can be loaded into the PEG matrix. It should be noted that ALD processing can also be done at lower temperatures (below 50 °C) where thermal stability of the drug is less critical. During the 150 °C process described here, we did not observe any decomposition of the Fluorescein. Since the fluorescein itself is driven through the membrane by electrostatics and by diffusion, we propose that substances of similar size and charge as the fluorescein will behave in a comparable manner. It is difficult to judge how the release functionality would perform for substances that are substantially larger, such as proteins, or neutral or positively charged molecules. Future work is needed to fully characterize the delivery functionality for various molecules.

The amount of molecules that can be released from the hybridized samples is only depending on the concentration within the PEG and thus independent of the conducting polymer. In our study a concentration of 1 mg/ml was used, resulting in a maximal theoretical loading of 25 ng in the test-samples. From the primary polymer coated films, we were able to release 22 ng in average during subsequent activation of the gating layer which equals 88% of what was found to be theoretically loaded. From the samples featuring additionally the secondary polymer layer, we could measure a total release of 5 ng corresponding to 20% of the theoretical loading. We assume that the loss in the loading might have occurred during the fabrication, especially while immersing the sample in water during electro-polymerization of the secondary polymer. This loss could potentially be reduced by replacing the low molecular weight PEG with a higher molecular weight PEG, having a lower solubility, and thus would not be depleted at the same rate during the processing. Using such a polymer would, however, also have an influence on the release characteristics. While loss of drugs and long-term stability of the loaded substance can tentatively be improved, high dosage within short time frames will be limited by the dissolution rate of the high molecular weight PEG. Taking the high solubility of Fluorescein into account, which is an appropriate representative for therapeutic drugs with respect to size and charge, the loading capacity can easily be extended by a factor 100–200 at retaining the overall dimensions of the system.

In practice, several substances could even be loaded within the same PEG matrix to yield a multifunctional system. PEG is commonly used in various pharmaceutical products and should be inert toward a wide range of therapeutic compounds. If release of various substances is to be controlled independently, a more sophisticated PEG deposition process could be used to form patches containing the different drugs before hybridization. By introducing further process steps, such as patterning the PEDOT layer into conducting and non-conducting regions, the different drug patches could in addition be driven independently of each other. The variety of possible substances in combination with the possibility to use the system either in single dispense mode or as a multiple dispensing system, enables the use in manifold applications. This is further supported by the fact that the hybrid material was found to be biocompatible in the direct contact test. The cells displayed good viability prior to fixation and were densely spread over the sample surface. Close examination by SEM imaging revealed that the cells interconnect with the VPP polymer surface and are able to form a network further supporting that the surface is suitable as a culture substrate. Last, but not least, the system could be autoclaved for sterilization which strengthens its practical usefulness.

In summary, we introduced a novel hybridized organic-inorganic material that forms a simple, minimalistic and yet highly functional drug release system. An ALD process has been used for the first time to convert a liquid polymer into a solid material. This revolutionary approach permits storage of large amounts of various molecules in a micrometer thin film which makes our approach superior to commonly used bulky or complex systems with the same functionality. Optimal performance with respect to release characteristics was achieved by introducing a two-layer conducting polymer coating to the hybridized storage film. This combination enabled the controlled release of molecules using an electrical trigger signal so that expulsion of stored substances (in this case Fluorescein) could be actively controlled with high precision. Our approach is the first example of a new generation of simple, yet sophisticated release systems and shows great promise for use in biomedical or lab-on-chip applications in which controlled dosage of various substances with high precision is of interest.

## Methods

### Porous Si formation & spin-coating

A 4 inch Si (100) wafer was dipped into a solution containing 5.1 mL of HF (wt. 5%), 195 mL of H_2_O and 0.061 g of AgNO_3_ (Sigma Aldrich, 99.9999%) for 20 s under room light to deposit small Ag crystallites (40–200 nm in diameter) on to the exposed Si surface. Upon Ag deposition, the wafer was submerged into an etching solution of 50 mL HF (wt. 5%) and 0.4 mL H_2_O_2_ (wt. 31%) for 10 min to create the porous Si.

PEG 400, blended with the fluorescent model drug, was spin-coated at 6000 RPM for 30 s on a porous Si wafer, produced with the method explained above.

### Atomic Layer Deposition

The ALD ZnO layer was deposited in a vertical flow hot wall reactor (OpAL - manufactured by Oxford Inst, Britain) through sequential cyclic reactions between diethylzinc (Strem Chemicals Inc.) and deionized water at 150 °C with 40 ms and 20 ms dose durations respectively. Following each dose step, the sample was further exposed to the precursor by flooding the exhaust line for 10 s with 400 sccm of Ar allowing reactants to diffuse into the PEG layer. 25 sccm of N_2_ was used as the carrier gas. The chamber was kept between 180–350 mTorr during the process. Between each ALD cycle, the reactor was purged for 15 s to remove remaining reactants and byproducts from the deposition environment to prevent any parasitic CVD reactions. Under these conditions, the ZnO growth rate was determined to be approx. 0.2 nm/cycle.

### PEDOT Deposition

Hybridized films were covered with a conducting polymer layer for controlled release of incorporated molecules. An initial vapor-phase polymerization (VPP) step was performed to yield a conducting surface for subsequent electro-polymerization of a secondary release layer. A 40% solution of Fe_3_(TOS) in butanol with pyridine in a molar ratio of 2:1 was prepared according to Winther-Jensen *et al.*^32^. This solution was applied to the hybridized PEG/ZnO layer by the casting method shown in Fig. 3b. The oxidizing layer was dried at 60 °C for 5 min and subsequently transferred to the vapor-phase deposition chamber providing the EDOT-monomer vapor at a pressure of 10 mbar (Fig. b,c). Polymer formation was conducted over 5 hours at room temperature for complete conversion of the oxidant and the monomer to a polymer film with a thickness of roughly 1 μm.

A fraction of samples having the primary polymer layer were extracted from further processing while the remaining were mounted in a Teflon-cell for electrochemical deposition of a thicker conducting polymer coating to realize a well-defined release on demand behavior. Electrical connection to the primary polymer sample was done using silver paint and a crocodile clamp at the edge of the sample. The secondary polymer was deposited under potentiostatic conditions (0.9 V) in a conventional three-electrode setup from an aqueous solution of 3,4-ethylenedioxythiophene (EDOT) and poly(sodium 4-styrenesulfonate) (NaPSS) at a concentration of 0.01 M and 5 mg ml^−1^ respectively. Growth of the film was controlled by a potentiostat (Metrohm Autolab) and deposition charge (100 mCcm^−2^) was used for approximation of the layer thickness.

### Scanning Electron Microcopy

Samples were investigated with a high-resolution scanning electron microscope (Nova NanoSEM) manufactured by FEI with a nominal maximum resolution of 1 nm.

### X-Ray Photoelectron Spectroscopy

XPS analysis was performed with a Perkin Elmer PHI 5600 ESCA system under ultra-high vacuum conditions with a tilt angle of 45°. The X-ray source was Mg (X-ray voltage: 13 kV). The anode power was set at 300 W. The analysis was performed between 0–1200 eV with a pass energy of 187.85 eV. The step voltage and duration were 0.8 eV and 20 ms. The depth profile was constructed by sputtering the sample (area: 2 × 2 mm) with Ar atoms (ionization energy: 200 eV) in steps of 1 minute before performing XPS analysis at each depth (repeated 50 times to construct a depth profile). In this case, the voltage step and duration were set to 0.025 eV and 100 ms for higher precision. The evaluation was performed using the software: MultiPak Version 9.4.0.7, 2012-11-27.

### Analytical release measurement

Release functionality of the hybridized samples was assessed by monitoring the elution of a model substance, here fluorescein sodium salt, at various sample configurations. Therefore small pieces of coated and uncoated samples were mounted in a release-cell, confining a defined exposed area (4 mm diameter) and allowing a small test volume of one milliliter saline solution (PBS, 0.01M) as electrolyte. A potentiostat was connected in the three electrode setup with the sample as working electrode, a Pt-foil as counter electrode and an Ag/AgCl reference electrode. A positive potential (+0.5 V) was used to suppress the release and a negative potential (−0.5 V) to trigger an active release (see Fig. 5a). The cumulative fluorescence intensity of the electrolyte during the release experiment was repeatedly measured in an Enspire Envision Platereader (Perkin Elmer) using an excitation signal at 475 nm and observing the emission at 512 nm. LOD and LOQ values for the Fluorescein detection were 0.18 ng and 0.59 ng respectively and the calibration curve was linear between 0.5 ng and 100 ng. For ease of comparison, intensity values in Fig. 5a are normed for passive (no potential applied) and active measurements. Multiple dosage attempts for repeatability testing were performed using repetitive blocks of 2 min of release potential, followed by 10 min (6 min in Fig. 5b) under suppressing conditions. Considering the high water solubility of Fluorescein (500 mg ml^−1^) and the drug-loading within our test-samples (25 ng), sink conditions can be assumed to maintain during the entire measurement time.

### Optical release experiment

Polymer coated hybrid samples were placed under a stereomicroscope equipped with a UV light source (Nikon Intensilight C-HGFI) and filter (GFP-B bandpass filter, MNE44100, Nikon) for visual inspection of the fluorescein release behavior. The miniaturized three electrode setup described in the previous section was used and release triggering was performed for 2 min at −0.5 V followed by a suppressing state (+0.5 V) for 4 min. Images were taken at the end of the suppressing state (t: 0 min and 8 min) as well as at the end of the activated release period (t: 2 min and 10 min) according to Fig. 5d.

### Biocompatibility tests

Primary polymer coated hybrid samples were tested for biocompatibility by means of a direct contact test using the human neuroblastoma cell line SH-SY5Y, purchased from ATCC. Cells were routinely cultured in a 1:1 mix of Eagle’s Minimum Essential Medium and Ham’s F12 with addition of 2mM Glutamine (Sigma D8062), MEM Non-essential Amino Acid solution (Sigma M7145), 15% Fetal Bovine Serum (Sigma F0804) and Penicillin-Streptomycin (Sigma P4333). Medium was exchanged every 2–3 days. Subcultivation was performed at confluency, 1–2 times per week, using enzymatic treatment with TrypsinEDTA (SigmaAldrich T3924), and at least twice after defrosting before experiments.

The hybrid samples were sterilized in an autoclave (121 °C, 20 min) after the VPP polymerization and cells were subsequently seeded on top of these probes in full culture medium. After an incubation time of 24 h, the cells were rinsed twice with PBS and exposed to a fixation solution (2.4% Formaldehyde solution +10% Glurataldehyde solution in PBS) for 1 h at 4 °C. Following the fixation procedure the cells were washed twice with H_2_O and subsequently dehydrated in an ascending ethanol series (30%, 50%, 60%, 70%, 80%, 90% and 100%) by exposing the cells for 5 mins to each solution. For SEM imaging, the samples were covered with a 5 nm sputtered gold layer.

### Reagents

Polyethyleneglycol (PEG400), Fluorescein sodium salt, Fe_3_(TOS), 3,4-ethylenedioxythiophene (EDOT), poly(sodium 4-styrenesulfonate) (NaPSS), 1-butanol, Phosphate buffered saline (PBS, 0.1 M), Formaldehyde solution (≥36.0% in H2O), Glutaraldehyde solution and Ethanol were purchased from Sigma Aldrich and unless otherwise specified used without further purification. Ultrapure water (18.2 MΩ∙cm) was prepared using a standard Milli-Q system.

## Additional Information

**How to cite this article**: Boehler, C. *et al.* A Simple Approach for Molecular Controlled Release based on Atomic Layer Deposition Hybridized Organic-Inorganic Layers. *Sci. Rep.* **6**, 19574; doi: 10.1038/srep19574 (2016).
